# The Effects of Exercise Induced Muscle Damage on Knee Joint Torque and Balance Performance

**DOI:** 10.3390/sports6030101

**Published:** 2018-09-19

**Authors:** Nicole C. Dabbs, Harish Chander

**Affiliations:** 1 Biomechanics Laboratory, Department of Kinesiology, California State University, San Bernardino, CA 92407, USA; 2 Neuromechanics Laboratory, Department of Kinesiology, Mississippi State University, Starkville, MS 39759, USA; hchander@colled.msstate.edu

**Keywords:** balance, stability, torque, muscle soreness

## Abstract

The purpose of this investigation is to determine the effects of exercise induced muscle damage (EIMD) on balance and knee joint torque. Thirteen males and females volunteered to participate in the study. Following a familiarization session, baseline measures were obtained for isometric torque measured during a maximal voluntary isometric contraction (MVIC) for knee flexors and extensors, and ankle dorsi-flexors and plantar-flexors. Additionally, balance performance was tested in double leg (DL), and right single leg (RSL) static and dynamic unstable stability was measured. Participants then performed the muscle damage protocol of front loaded Bulgarian split squats. All measurements were re-assessed for torque and balance immediately and up to 72 h afterwards. A one-way repeated-measures analysis of variance (ANOVA) was used to analyze differences between baseline and all time-points for torque and balance measures. There was a significant time effect for knee extensors MVIC torque, where baseline measures are greater than post EIMD, 24 h and 48 h post EIMD. There was no significant time effect for all balance conditions. These results provide evidence of EIMD following high intensity eccentric exercises with significant reductions in knee extensor torque up to at least 48 h and show that balance was not compromised following EIMD.

## 1. Introduction

Exercise induced muscle damage (EIMD) results from unaccustomed exercise or by performing an exercise that is of high intensity and/or duration [1,2]. It is well accepted that the presence of EIMD negatively impacts an individual’s performance [2]. Previous literature has analyzed the time course of changes after maximal exercise induced muscle damaging protocols, especially assessing individual performance, prior to and immediately after EIMD, as well as 24 h, 48 h and 72 h post-EIMD [3,4]. The presence of EIMD can be established by analyzing indirect indicators such as blood markers and analyzing an exercise performed by the individual. The American College of Sports Medicine’s (ACSM) health-related components of fitness, which include cardiorespiratory fitness, body composition, flexibility, muscular strength and endurance, have generally been used to assess an overall fitness level. Additionally, ACSM’s skill-related components of fitness, which include agility, coordination, power, speed, reaction time and balance [5], have also been used to assess fitness levels that are specific to a particular skill or task. Of these skill-related components, balance is an integral part of an individual’s performance, which involves the upright maintenance of the body for effective and successful completion of athletic, recreationally trained, sporting, occupational or any daily living task [6].

The maintenance of upright balance is a complex task governed by the postural control system, which involves intact sensory and motor neuromuscular systems [7] that work together to keep the body’s center of gravity within the base of support [8]. However, degradation of any of these neuromuscular systems can negatively impact balance [9] and potentially lead to poor skill performance. One such degradation is EIMD, which results from a physically exerting exercise, especially involving unaccustomed, high intensity and duration or eccentric exercise [1,2]. When these neuromuscular systems are degraded or defective, there is an increased demand on the body’s postural control system, and the body’s postural sway increases [10,11]. Assessment of balance in various sensory conditions, such as with and without vision while standing on a stable or unstable surface either in bilateral or unilateral stance [12], challenges the postural control system and tests its integrity. An increase in postural sway is identified as a lowered balance performance, which subsequently has been related to an increased risk of falls and fall related injuries [13].

Previous studies have also well documented the impact of EIMD on neuromuscular function. Exercise induced muscle damage results in an immediate and prolonged reduction in force and power generation capacity from the muscle as well as an increased physiological demand [2]. Additionally, specific skills such as cycling and vertical jumping performance have also been shown to be inhibited due to EIMD [2]. An isometric measure of joint torque during a maximal voluntary isometric contraction (MVIC) has been used as a reliable measure to identify the presence of EIMD, and a reduction in peak joint torque has been used as evidence of EIMD [14]. The impact of localized muscular fatigue after a strenuous workload on balance performance has been studied extensively with decrements in balance performance reported following acute localized muscular fatigue [15,16,17,18,19]. One of the primary reasons for this decrease in balance performance is the decrease in proprioceptive and somatosensory feedback due to muscular fatigue in balance maintenance [16,17,19]. Subsequently, a decreased proprioception has also been reported after EIMD [2]. Additionally, balance performance on athletes have been an area that is well emphasized [6,20,21,22,23,24]. However, there is still a dearth of literature on the impact of EIMD on balance, a vital skill-related component of fitness, and specifically on the changes in balance performance over the time course of EIMD on a recreationally trained population. Such information, though, would be important for practitioners to have if their client’s balance would be compromised when prescribing exercises following high intensity training to minimize the risk of injury. Therefore, the purpose of the study was to assess the effects of EIMD on knee joint torque and balance performance in recreationally trained individuals, prior to and after EIMD; specifically, along the time course changes of EIMD (immediately post EIMD, 24 h, 48 h and 72 h post EIMD). The authors hypothesized that knee joint torque and balance performance would be lowered after EIMD and gradually improve over the three-day testing period.

## 2. Materials and Methods

### 2.1. Participants

Thirteen recreationally trained males (*n* = 5) and females (*n* = 8) (means ± standard deviation; age = 20 ± 1 year; height = 161.49 ± 8.88 cm; weight = 55.82 ± 18.41 kg) volunteered to participate in a five-session protocol that was approved by the university Institutional Review Board and meets the ethical standards in sport and exercise science research [25]. Recreationally trained individuals were defined as participating in resistance training 3–5 times per week. Any participant with a recent history (in the last 6 months) of lower body musculoskeletal or orthopedic injury or who was taking medications known to alter balance, musculoskeletal, or central nervous system functions relating to posture and motor control was excluded from participating. Individuals taking prescription pain and/or psychiatric medications were also excluded. In addition, we screened participants by a questionnaire for potential risk factors to the exercise protocol (e.g., rhabdomyolysis). We asked participants to not perform any lower body exercise or take any pain medications throughout the study, to keep food and water intake consistent, and to refrain from caffeine consumption for 8 h prior to each testing session.

### 2.2. Experimental Design

A repeated measures design was implemented with one familiarization day that included informed consent, anthropometrics, and familiarization of all experimental protocols. Following the familiarization session, participants visited the laboratory for testing on 4 consecutive days. All baseline measures were assessed for balance and right knee and ankle joint torque (explained under measures) on day 1 following a dynamic warm-up. The right leg only was tested for torques and single leg balance measures, to keep consistency between subjects. The dynamic warm-up consisted of 2 sets of 15 m jogging, leg swings, high knees, exaggerated lunges, and walking planks. After baseline measures were taken, participants completed the exercise induced muscle damage protocol, which consisted of split squats using a Jones Machine^®^ (BodyCraft Inc., Lewis center, OH, USA) by performing 3 sets to volitional failure on each leg. The Jones Machine^®^ was front loaded with 40% of their body weight. During the split squat, the back leg was placed on a padded bench for support and the knee was placed into 90° of flexion, allowing focus on single leg performance of the front leg. We provided assistance on the concentric phase after the participants reached 90° of knee flexion of the front knee on the exercising leg, allowing greater focus on the eccentric phase [26,27]. Immediately following EIMD protocol, participants were re-assessed for balance and torque measurements. Participants returned to the laboratory 24 h, 48 h, and 72 h post EIMD to evaluate measurements over time. Total volume-load (total reps × load) was calculated for each leg from the EIMD protocol.

### 2.3. Measures

#### 2.3.1. Knee and Ankle Joint Torque

Isometric torque values were assessed using the BioDex Isokinetic dynamometer (System 4 Pro™, Shirley, NY, USA). Maximal voluntary isometric contractions were performed for right knee extensors (quadriceps), knee flexors (hamstrings), ankle dorsiflexors; tibialis anterior (TA) and ankle plantar flexors; gastrocnemius (GM) with 3 trials of 3 s each. Participants were seated with the hip at 90° of right leg flexion and knee fixed in a flexed position at an angle of 60° below horizontal for quadriceps (QUAD) and hamstring (HAM) measurement. For right ankle joint torque, participants were seated with the hip at 90° of flexion and right ankle in a fixed position at an angle of 45°. Participants were instructed to produce maximal amount of force for each trial and were verbally encouraged during contractions. Maximum torque from the 3 trials was used for analysis for each muscle. 

#### 2.3.2. Balance

Static and dynamic balance was assessed using Biodex Balance System SD (model # 950–440, Balance System™ SD, Shirley, NY, USA). Each participant performed bilateral double leg (DL) static stability, DL dynamic unstable surface stability (level 4), unilateral right single leg (RSL) static stability, and RSL dynamic unstable surface stability (level 4) balance test. Anterior/Posterior (AP) sway, Medial/Lateral (ML) sway, and an overall sway scores were used for analysis for each balance test. Participants performed all balance tests without shoes on and hands next to their side. Each test consisted of three 20 s trials, and those trials were averaged for analysis. The Biodex Balance System SD ranges from levels 12 to 1 for dynamic balance (12 = most stable and 1 = least stable); level 4 is commonly used in balance assessment with active individuals. Smaller sway values represent more balance and postural stability, and larger sway values represent less balance and postural stability.

### 2.4. Statistical Analysis

A one-way repeated-measures analysis of variance (ANOVA) test was used to determine changes from baseline in balance and muscle pain over time. If main effects for time occurred, they were followed up with Bonferroni post-hoc analyses for pairwise differences with an adjusted alpha for comparisons from baseline. A paired-sample t-test was used to determine differences in total volume-load between left and right leg during EIMD protocol. All analyses were conducted using IBM SPSS™© software (SPSS 24, Inc., Chicago, IL, USA). Statistical significance was determined as *p* < 0.05.

## 3. Results

### 3.1. Total Volume-Load

There were no significant (*p* = 0.60) differences between left leg (1470.06 ± 503.07 kg) and right leg (1393.46 ± 340.53 kg) total volume-load during the EIMD protocol.

### 3.2. Torque Measures

**Figure 1:**
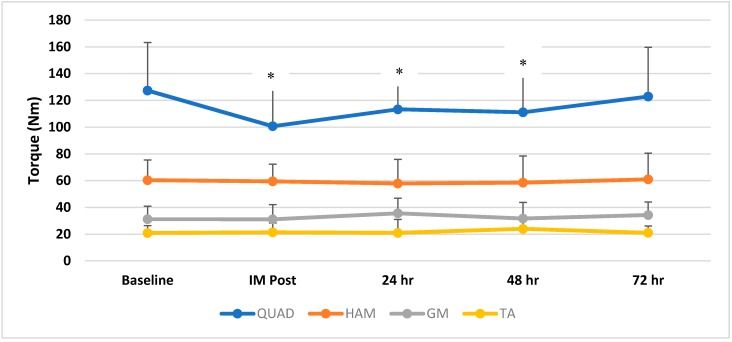
Mean and standard deviation for maximum quadriceps (QUAD), hamstring (HAM), gastrocnemius (GM) and tibialis anterior (TA) torque from baseline to immediate post (IM Post), 24 h post, 48 h post, and 72 h post. * indicates significant (*p* < 0.05) differences from baseline. Baseline QUAD torque was greater than IM post, 24 h post, and 48 h post. There were no significant differences (*p* > 0.05) between baseline and 72 h post in QUAD and throughout the study for all other variables.

There was a significant (*p* = 0.003, effect size = 0.28) time effect for QUAD MVIC torque, where baseline measures were greater than post EIMD (*p* = 0.001), 24 h post (*p* = 0.003), and 48 h post (*p* = 0.02) (Figure 1). There was no significant (*p* = 0.79; effect size = 0.03) time effect for HAM MVIC torque from pre (mean = 59.35, s = 14.74 Nm) to immediate post (IM post) (mean = 58.63, s = 12.48 Nm), 24 h post (mean = 57.18, s = 17.97 Nm), 48 h post (mean = 57.72, s = 19.89 Nm), and 72 h post (mean = 59.86, s = 19.23 Nm). There was no significant (*p* = 0.38; effect size = 0.08) time effect for GM MVIC torque from pre (mean = 31.16, s = 9.72 Nm) to IM-post (mean = 31.07, s = 11.01 Nm), 24 h post (mean = 35.56, s = 11.28 Nm), 48 h post (mean = 31.67, s = 12.11 Nm), and 72 h post (mean = 34.23, s = 9.84 Nm). There was no significant (*p* = 0.46; effect size = 0.07) time effect for TA MVIC torque from pre (mean = 20.84, s = 5.49 Nm) to IM-post (mean = 21.33, s = 7.03 Nm), 24 h post (mean = 20.95, s = 10.13 Nm), 48 h post (mean = 23.92, s = 8.36 Nm), and 72 h post (mean = 20.98, s = 5.15 Nm).

### 3.3. Balance Measures

There were no significant time effects for RSL static balance in overall sway (*p* = 0.17; effect size = 0.12), AP sway (*p* = 0.15; effect size = 0.12), and ML sway (*p* = 0.86; effect size = 0.02). There were no significant time effects for RSL dynamic unstable balance in overall sway (*p* = 0.08; effect size = 0.15) and ML sway (*p* = 0.31; effect size = 0.09). There was a significant (*p* = 0.03; effect size = 0.19) time effect for RSL dynamic unstable balance in AP sway; however, post-hoc analysis revealed no significant differences from baseline to IM post (*p* = 0.16), 24 h (*p* = 0.84), 48 h (*p* = 0.15), and 72 h (*p* = 0.05) post EIMD. There was no significant time effect for DL static balance in overall sway (*p* = 0.08; effect size = 0.15), AP sway (*p* = 0.12; effect size = 0.13), and ML sway (*p* = 0.38; effect size = 0.08) following EIMD. There was no significant time effect for DL dynamic unstable balance in overall sway (*p* = 0.51), AP sway (*p* = 0.22), and ML sway (*p* = 0.72) following EIMD (Table 1).

## 4. Discussion

The purpose of the study was to assess the impact of EIMD on knee and ankle joint torque and balance performance in recreationally trained individuals. The results from the study demonstrate significantly decreased torque production after an exercise induced damaging protocol but limited to only the knee extensor muscle. The results from the balance measures demonstrated no significant differences due to EIMD across all time points of measurements. The statistically significant differences in torque measurements followed a timeline of 72 h after EIMD that has been well understood and reported previously [1,2,28]. The significant decline in knee joint torque was evident immediately after EIMD and at 24 h and 48 h post EIMD, but not significantly different from baseline at 72 h post EIMD. The balance performance was trended toward significance in a reduction at 48 h and 72 h, in comparison to baseline and immediately post muscle damaging protocol.

The impact of EIMD on athletic performance measures has been well studied previously. Athletic performance measures such as running, sprinting, cycling, and jumping, and subsequently the magnitude of power generation during these tasks due to EIMD, have been reported previously [1,2,28], with EIMD linked to detrimental athletic performance. The reductions in power generation or athletic performance have been shown immediately after the EIMD [2]. Similarly, force or torque generation has also been shown to be affected immediately after induced muscle damage [2]. Force or torque loss due to muscle damage can be as high as 50–65% from pre-exercise values with high intensity eccentric exercises [1]. The results from the study support previous literature as a significant reduction in quadriceps knee joint torque was seen immediately following the muscle damaging protocol which continued to be significantly lower during the 24-h and 48-h testing periods, providing evidence of muscle damage [1,28]. The initiation of the recovery process of generating optimal torque at 72 h also supported previous literature in the timeline of EIMD [2,28]. Physiologically, the stretching of the muscle during the eccentric muscle action, resulting in linear deformation and z-line streaming of the sarcomeres, has been shown to cause force/torque reduction due to EIMD. Damage to the soft series elastic components within the muscle and tendon attachments of these muscles has also been suggested as a reason for loss of force generation [1]. However, these significant differences in torque generation were only seen in the knee extensor (quadriceps) muscle, and not in the knee flexors and ankle musculature. The nature of the exercise performed for the muscle damaging protocol could explain why the torque reduction was evident only in the knee extensor muscle but not in other lower extremity muscles tested. The split-squats exercise predominantly works the knee extensor muscle, especially with the eccentric phase emphasized in this protocol. Additionally, the 40% body weight and the repetitions until volitional failure sets aided in damaging the knee extensor muscles significantly more.

While the impact of EIMD on athletic and sporting tasks is well studied and reported, an individual’s balance performance after EIMD, which is critical to successfully and efficiently perform athletic or sporting tasks, needs to be further explored. The results from the study demonstrated that balance performance was not altered after EIMD. The amount of muscle activity from the lower extremity muscles required to maintain an erect stable static stance is very minimal. However, the presence of muscle fatigue or damage can negatively impact the afferent and efferent postural control systems, resulting in a decline in balance performance [29]. Localized muscle fatigue or damage can negatively impact the postural control system by diminishing the role of proprioceptors in relaying joint position to the central nervous system [19] with alterations in the afferent somatosensory feedback and efferent motor unit firing rates [16,17,18,29] to maintain balance. Consequently, with altered sensory and motor systems resulting from muscle fatigue or damage, postural sway increases in balance tasks [16,17,18,29]. Additionally, with the greater postural control demands that exist with standing on unstable surfaces that compromise the proprioceptive feedback, the addition of muscular damage can further increase postural sway and be more detrimental to balance maintenance [16,17,18,29,30,31,32]. However, in the current study, balance performance in both static and dynamic testing conditions was not impacted by EIMD. The postural control of an individual is not only dependent upon the afferent proprioceptive and somatosensory system but also reliant on the afferent visual and vestibular systems that are not essentially affected by localized muscular fatigue [6,30]. Hence, balance performance during bilateral and unilateral (right leg) stance on stable and unstable support surface suggests that the visual and vestibular systems might have provided the necessary sensory feedback to maintain balance, rather than the proprioceptive/somatosensory system which is impacted by muscular fatigue or damage [6,9,15,18,29,30]. In the current study, balance performance did not decline, as the visual and vestibular systems of postural control were not challenged in maintaining balance. Hence, the findings from the study suggest that balance performance is not impacted by EIMD during static and dynamic balance measurements. However, these findings should be used with caution as balance measurements were performed in eyes-open condition when the visual system was not challenged.

Limitations to the study include a small sample size. However, all participants were tested on four consecutive days following a repeated-measures design, aiding in a greater statistical power and providing insights into the timeline of EIMD and its impact on balance and torque. Specifically, balance measures after EIMD, should be performed in testing conditions that challenge the entire postural control system. Using balance machines such as the NeuroCom Equitest and testing balance using the sensory organization test (SOT), which has the capability to test the three afferent systems (visual, vestibular and somatosensory/proprioceptive) individually [6], can help identify existing balance decrements, especially after muscular damage or fatigue. Additionally, participants in this study were recreationally trained, and not elite/athletic trained. However, this represents the majority of the population who can potentially be exposed to EIMD and, unlike the elite athletic population, they do not have immediate and constant rehabilitation care. Hence, the impact of EIMD on a recreationally trained population needs to be well understood, and this study adds to previous literature by demonstrating significant differences in torque generation but not balance. While there has been previous research that has compared balance performance between dominant and non-dominant limbs, most of the research indicates that there are no significant differences between the dominant and non-dominant limbs, with balance performance being symmetrical [20,33,34] and with no differences between preferred and non-preferred limbs [35]. Consequently, the aim of this current project was to determine if EIMD impacted balance performance over the 72-h time-period, and, in order to aid the feasibility of the project, only the right limb was used for balance testing. This is another limitation of the present study and future studies can be recommended for bilateral testing with different types of EIMD protocols.

## 5. Conclusions

The impact of EIMD on torque generation capability from the lower extremity muscles and on balance performance over the course of 72 h from the induction of muscle damage was analyzed in this study. Significant reductions in torque from the knee extensor muscle was evident immediately following the muscle damaging protocol and was significantly reduced until 48 h, providing evidence of muscle damage. Balance performance was not affected after muscle damage, suggesting that the visual and vestibular system might have provided the necessary sensory feedback to compensate for the altered feedback from the proprioceptive system due to muscle damage. Findings from the study help us understand the impact of EIMD on athletic performance and provide the time-course of changes in torque production and balance variables until 72 h after muscle damage. This information could be helpful in planning a return to exercise or rehabilitation for individuals who are recreationally trained and exposed to EIMD.

## Figures and Tables

**Table 1:** Mean ± SD of Balance Sway Index in DL (double leg) and RL (right leg) conditions. AP = Anterior/Posterior; ML = Medial/Lateral; IM = immediate; EIMD = exercise induced muscle damage. No significant differences from baseline in all balance measures.

Balance Condition	Balance Direction	Baseline	IM Post	24 h Post	48 h Post	72 h Post
**Static DL**	**Overall**	0.35 ± 0.15	0.66 ± 0.59	0.81 ± 1.18	0.45 ± 0.51	0.47 ± 0.37
	**AP**	0.26 ± 0.14	0.53 ± 0.54	0.73 ± 1.17	0.40 ± 0.52	0.34 ± 0.24
	**ML**	0.16 ± 0.09	0.25 ± 0.27	0.22 ± 0.22	0.17 ± 0.13	0.22 ± 0.28
**Static RL**	**Overall**	1.57 ± 1.10	1.37 ± 0.95	1.25 ± 0.73	1.26 ± 0.78	1.04 ± 0.56
	**AP**	1.10 ± 1.09	0.92 ± 0.90	0.83 ± 0.50	0.76 ± 0.43	0.61 ± 0.29
	**ML**	0.91 ± 0.53	0.88 ± 0.57	0.83 ± 0.57	0.86 ± 0.72	0.73 ± 0.56
**Unstable DL**	**Overall**	1.38 ± 0.72	1.55 ± 1.52	1.39 ± 0.84	1.28 ± 0.84	1.21 ± 0.71
	**AP**	1.13 ± 0.63	1.32 ± 1.26	1.12 ± 0.70	0.91 ± 0.55	0.96 ± 0.58
	**ML**	0.57 ± 0.41	0.70 ± 0.73	0.57 ± 0.51	0.66 ± 0.64	0.56 ± 0.38
**Unstable RL**	**Overall**	2.21 ± 1.27	2.14 ± 1.24	1.94 ± 0.98	1.72 ± 1.25	1.59 ± 0.85
	**AP**	1.37 ± 0.91	1.6 ± 0.93	1.33 ± 0.74	1.04 ± 0.65	1.02 ± 0.49
	**ML**	1.57 ± 1.02	1.36 ± 0.84	1.14 ± 0.87	1.18 ± 1.18	1.11 ± 0.65
